# Exercise rehabilitation on home-dwelling patients with Alzheimer's disease - a randomized, controlled trial. Study protocol

**DOI:** 10.1186/1745-6215-11-92

**Published:** 2010-10-06

**Authors:** Kaisu H Pitkala, Minna M Raivio, Marja-Liisa Laakkonen, Reijo S Tilvis, Hannu Kautiainen, Timo E Strandberg

**Affiliations:** 1 Unit of General Practice, Helsinki University Central Hospital, University of Helsinki, PO Box 20, 00014 University of Helsinki, Finland; 2 The Social Insurance Institution of Finland, Helsinki, Finland; 3 Helsinki City Health Center, Laakso Hospital, Lääkärikatu 8, 00250 Helsinki, Finland; 4 Department of Internal Medicine and Geriatrics, Helsinki University Central Hospital, PO Box 340, 00029 HUS, Finland; 5 Orton Orthopedic Hospital, Orton Foundation, Helsinki, Finland; 6 Unit of Family Practice, Central Finland Central Hospital, Jyväskylä, Finland, Hämeentie 1, 44150 Äänekoski, Finland; 7 Institute of Health Sciences/Geriatrics, University of Oulu, Oulu, Unit of General Practice, PO Box 5000, FIN-90014 Oulu, Finland

## Abstract

**Background:**

Besides cognitive decline, Alzheimer's disease (AD) leads to physical disability, need for help and permanent institutional care. The trials investigating effects of exercise rehabilitation on physical functioning of home-dwelling older dementia patients are still scarce. The aim of this study is to investigate the effectiveness of intensive exercise rehabilitation lasting for one year on mobility and physical functioning of home-dwelling patients with AD.

**Methods:**

During years 2008-2010, patients with AD (n = 210) living with their spousal caregiver in community are recruited using central AD registers in Finland, and they are offered exercise rehabilitation lasting for one year. The patients are randomized into three arms: 1) tailored home-based exercise twice weekly 2) group-based exercise twice weekly in rehabilitation center 3) control group with usual care and information of exercise and nutrition. Main outcome measures will be Guralnik's mobility and balance tests and FIM-test to assess physical functioning. Secondary measures will be cognition, neuropsychiatric symptoms according to the Neuropsychiatric Inventory, caregivers' burden, depression and health-related quality of life (RAND-36). Data concerning admissions to institutional care and the use and costs of health and social services will be collected during a two year follow-up.

**Discussion:**

To our knowledge this is the first large scale trial exploring whether home-dwelling patients with AD will benefit from intense and long-lasting exercise rehabilitation in respect to their mobility and physical functioning. It will also provide data on cost-effectiveness of the intervention.

**Trial registration:**

ACTRN12608000037303

## Background

Dementia is the most important disease group leading to disability and requiring social and health care among older individuals [1]. The most prevalent of dementing illnesses (55-80%) is Alzheimer's disease which leads to gradual and often steady decline of cognitive functioning, to various behavioural and psychological symptoms and physical disability [2]. Disabilities lead to loss of autonomy, require help and support as well as institutional care. Dementia with its consequences is also very expensive for the society [1].

One pivotal problem in dementia leading to need for help and institutional care is decline in mobility. Locomotion gets stiff, walking decelerates and falls are common [3]. The stiffness and emerging disabilities makes caregivers' work demanding. Going outdoors, getting dressed and undressed as well as transfers from one place to another become challenging [1]. This process is accelerated by patient's progressive sarcopenia, weight loss and muscular weakness which impede body control and predisposes to falls [4].

Some earlier studies have suggested that physical exercise may be beneficial in dementia. Physical activity and regular exercise training may slow down cognitive decline [5,6], and it has positive effects on cognition among those with cognitive decline [7]. Exercise alleviates depression and reduces behavioural symptoms in dementia patients [7,8]. There are several controlled trials testing effectiveness of endurance or strength training among patients with dementia or in samples with a large proportion of patients with dementia. Most of the trials have a low number of participants and, therefore, low power to show effectiveness. In addition, many of the trials are not randomized. A meta-analysis of these trials showed a small but significant effect on strength and endurance outcomes [9]. Majority of these trials have been performed in nursing homes or in long-term care. One randomized study in a nursing home setting has shown that intensive, long-lasting exercise training may even improve physical functioning of Alzheimer's patients [10]. According to the trials showing effectiveness on physical functioning, the training program should be intensive, long-lasting and versatile.

We found only four clinical exercise trials targeted on home-dwelling patients with dementia [8,11-13]. Three of them were performed with very low number of patients (N = 29-81) [11-13] and two of them did not show any effectiveness [11,12]. Teri's study [8] was targeted to improve behavioural symptoms in dementia. Thus, there are still only few rigorous randomized trials investigating the effectiveness of physical rehabilitation among home-dwelling patients with dementia. Although some studies have shown promising effects of exercise on physical functioning among institutionalized patients with dementia, there are no such studies among home-dwelling patients with Alzheimer's disease (AD).

We are testing intensive exercise training performed twice weekly either at patient's home or in a day centre and lasting for one year on patients Alzheimer's disease (AD). The primary aim of this study is to investigate the effects of exercise on AD patients' mobility and physical functioning. The secondary aims are to assess the effects of exercise interventions on patients' neuropsychiatric symptoms and cognition. We also investigate the effects of intervention on the use and costs of health care services, admissions of AD patients to permanent institutional care (cost-effectiveness), falls and mortality. The effects on the caregivers' quality of life will also be assessed.

## Methods

### General design

This is a multicenter, controlled intervention trial with a prospective, randomized design. The aim is to study the effects of two exercise interventions, one performed at AD patients' home for one hour twice a week, and another performed at rehabilitation day centres for four hours twice a week. The interventions will last for one year, and they are compared with usual care. The study has been approved by the Ethics Committee of the Helsinki University Central Hospital. Informed consent has been obtained from each patient and/or their spousal caregiver before any study procedure which are performed according to good clinical practice. An executive committee (KHP, TES, RST) is responsible for the planning, conduct and monitoring of the study.

### Participants

The study was started in 2008 by posting a letter to a random sample of patients receiving reimbursement for AD medication according to the centralized Drug Imbursement Register of the Social Insurance Institution of Finland (Kela), and his/her spouse living at the same address. All these patients had undergone detailed diagnostic assessments before acquiring reimbursement for their AD medication (e.g. cognitive and neuropsychological tests, neuroimaging and laboratory tests). The outline of the exercise study was described, and they were invited to participate.

Besides AD and spouse requirement, the inclusion criteria are:

- living in the greater Helsinki area (Helsinki - Espoo - Vantaa)

- age over 64 years

- no terminal disease, no severe hemiparesis

- independent mobility - with walking devices if needed

- frailty: at least one fall during last year, slowed walking speed, or weight loss during one year

All the spouses of the AD patients showing interest in participation are sent a questionnaire to confirm the fulfilment of the inclusion criteria and they are further interviewed via telephone about the frailty criteria, mobility and diseases. Those couples fulfilling inclusion criteria are invited for the first study nurse visit. At the beginning of the first visit the couples are given written and oral information of the study and they are asked to sign an informed consent. Both patients and their spousal caregivers give consent. In case of the AD patient's poor capability of judgement the spousal caregiver gives proxy's consent for both spouses.

### Study procedures

The baseline study visit lasts about two hours and includes interview of both spouses' demographic data, diagnoses, medications used, and baseline use of health and social services. The diagnoses and medications are confirmed from medical records provided by the couples. Charlson comorbidity index is calculated to assess the severity and prognostic value of the participants' disease burden [14].

The AD patients are assessed by Clinical Dementia Rating Scale (CDR) [15], Minimental State Examination (MMSE) [16], verbal flow [17,18], clock drawing test [19], Mininutritional Assessment (MNA) [20], Neuropsychiatric Inventory (NPI) [21,22], Cornell depression test [23], mobility tests and balance tests [24], and the Functional Independence Measure (FIM) [25]. Patients' weight, blood pressure (with 10 min rest and recorded in a sitting position), heart rate and handgrip strength [26] are assessed on each visit. Falls and fractures are recorded with the aid of diaries kept by caregivers and recorded on each visit.

The spousal caregivers are assessed by the Zarit burden scale [27], Geriatric Depression Scale (GDS) [28], RAND36 [29,30] and mobility and balance tests [24]. At six months the caregivers are asked to give feedback for the study and their satisfaction is assessed.

The couples are randomized in clusters of 30. After assessing 30 couples with eligibility and frailty criteria confirmed and agreeing to consent the study, the randomization is performed. Couples are randomly allocated by means of computer-generated random numbers to three arms: to receive home rehabilitation, day care rehabilitation or to receive the usual care. The study nurse calls by telephone to a randomization staff member who has not seen the couples neither their clinical records. She assigns the next number from the computer and the group assignment to the patient.

Participating couples are assessed by two study nurses four times during the year: at baseline, and at 3, 6, and 12 months. In addition, those remaining in the community will be invited for a follow-up visit at 24 months. The study assessment procedures are described in table 1. Use of health and social services, institutionalizations and death dates on both the patients and the caregivers will be retrieved from the central registers until two years from baseline measurements.

**Table 1:** Study assessment procedures and timetable.

Assessment^1^	Telehone interview	Baseline visit	3 month visit	6 month visit	12 month visit	24 month visit
**AD patient**						
Inclusion criteria	X					
Demographics, diagnoses, drugs		X				
CDR		X				X
MMSE		X			X	X
Verbal flow, clock drawing test		X	X	X	X	X
MNA		X				
Weight, BMI		X	X	X	X	X
Mobility and balance tests by Guralnik		X	X	X	X	X
FIM		X	X	X	X	X
NPI		X		X		
Grip strength, blood pressure, heart rate		X	X	X	X	X
Cornell depression scale		X			X	
Falls and fractures			X	X	X	X
Use of health and social services, admission to permanent institutional care, mortality			X	X	X	X
Spousal caregiver						
Demographics, diagnoses, drugs		X				
Zarit burden scale		X			X	X
GDS		X			X	
RAND-36 QOL		X			X	X
Mobility and balance tests by Guralnik		X			X	
Use of health and social services, mortality			X	X	X	X

^1 ^CDR = Clinical dementia Rating Scale (Hughes et al 1982) (0.5 suggests possible dementia, 1 mild, 2 moderate, and 3 severe dementia); MMSE = Minimental State Examination (Folstein et al. 1972); MNA = Mininutritional Assessment (Guigoz et al. 2002) (< 24 suggests risk for malnutrition or malnourishment); BMI = Body mass index; Guralnik's mobility tests (Guralnik et al. 1994); FIM = Measure of functioning (Pollak et al. 1996); NPI = Neuropsychiatric inventory (Cummings et al. 1994, 1997); Cornell = depression test (Alexopoulos et al. 1988); Zarit burden scale (Zarit et al. 1980), GDS = Geriatric depression scale (Yesavage et al. 1982-3); RAND-36 = Measure of health related quality of life (Parker et al. 2006)

**Figure 1:**
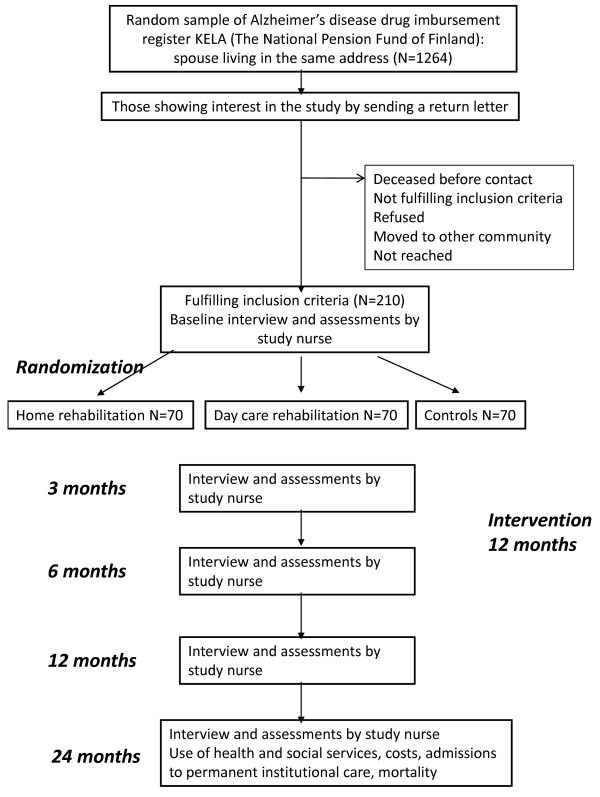
**Flow chart of the study**.

The flow chart of the study is described in figure 1.

### Intervention

A total of 210 patients will be randomized in three groups (70 per each arm) as follows:

#### Intervention

A geriatrician performs comprehensive geriatric assessment prior the intervention and makes the rehabilitation plan including also nutritional care.

1. Home-based physical exercise and rehabilitation procedures (one hour twice a week for 12 months) are performed by physiotherapy professionals of an expert organization (Suomen Fysiogeriatria), with long-term experience of dementia exercise rehabilitation. Interventions are tailored according to the patient's and caregiver's needs (for example: training transfers, stair climbing, balance training, executive functioning or dual-tasking).

2. Day rehabilitation centre-based physical exercise and rehabilitation (4 hours twice a week for 12 months) is organized and performed by professionals of dementia physiotherapy at two day rehabilitation centres (with gym and experience) performing the day care rehabilitation. Intervention consists of endurance, balance, and strength training as well as dual task training and exercises directed to improve executive functioning. Taxi transportation and lunch are provided for participants during the intervention days.

The intervention is provided up to one year or until patient is admitted to permanent institutional care.

Patients randomized to the control group receive the usual care in normal health care system, but they are provided with oral and written advice on nutrition and exercise by the study organization.

As a nutritional advice, all patients receive advice to ensure a sufficient intake of energy and protein [4] and to take 20 μg of vitamin D supplements per day [31].

### Outcome measurements

Primary outcome measures are changes in patients' mobility assessed by the tests described by Guralnik et al [24] and in physical functioning according to the FIM measure [25].

Secondary outcome measures are changes in patients' cognition by verbal flow [17] and MMSE [16], neuropsychiatric symptoms by NPI [21,22] and Cornell [23], number of falls, and permanent institutionalizations and time spent at home. Changes in caregivers' satisfaction, burden by the Zarit burden scale [27], depression by the GDS [28], health-related quality of life by the RAND-36 [29,30] will be measured as secondary outcomes.

Total mortality, use and costs of health and social care services of both spouses as well as cost-effectiveness of the intervention will be measured up to 24 months from the beginning of the baseline measurements.

### Statistical analyses

Sample size was calculated based on the FIM measure [25]. With an estimated standard deviation 20, and type I error 5%, 80% power, 63 patients would be needed in each group to show a 10-point difference between groups. With estimated 10% drop outs in each group about 70 patients are needed in each group. Data will be analyzed on intention-to-treat basis. Imputation method "the last observation carried forward" (LOCF) and "Worst-rank score"-principle will be used.

In these baseline findings, for the continuous variables, descriptive values were expressed by means with standard deviations (SD) and medians with range. For the variables with a normal (Gaussian) distribution, statistical comparisons between the groups are made by using an analysis of variance. If the variables have a non-normal distribution or ordinal level, statistical comparison between groups was made using the Kruskall-Wallis test. Measures with a discrete distribution were expressed as percentages (%) and analysed by Chi-Square or Fischer's exact test when appropriate.

## Discussion

This trial investigates the effects of intense, long-lasting physical exercise on home-dwelling patients with AD. It has two active intervention arms which are compared with usual care. One third of patients receive home-based tailored exercise twice a week one hour at the time for one year or until the patient is admitted to permanent institutional care. Another third of patients are receiving versatile exercise training including endurance, balance, and strength training as well as dual task training and exercises directed to improve executive functioning. This intervention is provided in day centers for four hours/day twice weekly for one year or until the patient is admitted to permanent institutional care.

There are several strengths in our study. All participants have an established diagnosis of AD because all are recruited from the drug register. This register requires definite diagnosis based on cognitive tests, disability, laboratory tests, and CT scan or MRI scan for the AD drug reimbursement. All participants suffer from frailty, and have therefore a need for exercise rehabilitation. Thus, the findings should be applicable in real life. The exercise training is intense and long-lasting, thus, reinforcing effectiveness of the intervention. The used resources and costs will be counted in detail.

However, there are also several challenges in this study. First, the population is old and frail with many comorbidities, and, thus, vulnerable to competing causes of complications and deaths. The second challenge relates to a sufficient difference to be attained between the groups with our intervention. Contamination of the control group is probably not a problem, because exercise rehabilitation is rarely available for dementia patients in Finland unless they are the Second World War veterans [32], in practice over 83 years of age in 2008. Although the FIM has been developed to measure outcomes of rehabilitation and validated among very old patients [25], it is not very clear how the FIM measure will respond to change in functioning as a consequence of exercise intervention in patients with AD. It was also challenging to find suitable scales to measure change in cognition in this heterogeneous group consisting of all stages of dementia.

To our knowledge, this is the first large scale intervention trial exploring the effects of exercise on physical functioning among home-dwelling patients with dementia. This study will provide data whether exercise will have effect on physical functioning and use of health services among home-dwelling AD patients at risk for functional decline and institutional care.

## List of abbreviations

AD: Alzheimer's disease; BMI: Body Mass Index; CDR: Clinical Dementia Rating scale; FIM: Functional Independence Measure; GDS: Geriatric Depression Scale; LOCF: Last observation carried forward; MMSE: Minimental State Examination; MNA: Mininutritional Assessment; NPI: Neuropsychiatric Inventory; QOL: Quality-of-Life; SD: Standard Deviation

## Competing interests

Minna Raivio has been working part-time for the Social Insurance Institution of Finland. All the other authors declare that they have no competing interests.

## Authors' contributions

Details of contributors: 1. Conception and design (KHP, TES, RST, MMR, MLL, HK); 2. Acquisition of data, and statistical analysis and interpretation of data (KHP, TES, HK) 3. Drafting the article or revising it critically for important intellectual content (KHP, TES, RST, MMR, MLL, HK); 4. Final approval of the version to be submitted (KHP, TES, RST, MMR, MLL, HK). KHP is the guarantor.
